# T. S. M. A. Appointments

**Published:** 1887-09

**Authors:** 


### TEXAS STATE MEDICAL ASSOCIATION.

President Burroughs has made the following appointments: "A committee of seven to draft a circular letter to the people setting forth the necessity and advantages of a law to regulate the practice of medicine in Texas," under a resolution by Dr. M. A. Taylor, at last meeting. Drs. M. A. Taylor, Frank Allen, C. H. Wilkinson, W. P. Burt, C. J. C. King, A. W. Pope and C. F. Paine.

